# Gastric cancer treated in 2002 in Japan: 2009 annual report of the JGCA nationwide registry

**DOI:** 10.1007/s10120-012-0163-4

**Published:** 2012-06-23

**Authors:** Atsushi Nashimoto, Kohei Akazawa, Yoh Isobe, Isao Miyashiro, Hitoshi Katai, Yasuhiro Kodera, Shunichi Tsujitani, Yasuyuki Seto, Hiroshi Furukawa, Ichiro Oda, Hiroyuki Ono, Satoshi Tanabe, Michio Kaminishi

**Affiliations:** 1 Department of Surgery, Niigata Cancer Center Hospital, 2-15-3 Kawagishi-cho, Chuo-ku, Niigata, 951-8566 Japan; 2 Department of Medical Informatics, Niigata University Medical and Dental Hospital, Niigata, Japan; 3 Department of Surgery, National Hospital Organization Tokyo Medical Center, Tokyo, Japan; 4 Department of Surgery, Osaka Medical Center for Cancer and Cardiovascular Diseases, Osaka, Japan; 5 Department of Surgery, National Cancer Center Hospital, Tokyo, Japan; 6 Department of Surgery, Nagoya University School of Medicine, Nagoya, Japan; 7 Department of Surgery and Science, Graduate School of Medical Science, Kyushu University, Fukuoka, Japan; 8 Department of Gastrointestinal Surgery, Graduate School of Medicine, University of Tokyo, Tokyo, Japan; 9 Department of Surgery, Sakai City Hospital, Sakai, Japan; 10 Endoscopy Division, National Cancer Center Hospital, Tokyo, Japan; 11 Endoscopy Division, Shizuoka Cancer Center Hospital, Shizuoka, Japan; 12 Department of Gastroenterology, Kitasato University School of Medicine, Kanagawa, Japan; 13 Department of Surgery, Showa General Hospital, Tokyo, Japan

**Keywords:** Gastric cancer, Nationwide registry, 5-year survival rate (5YEARS), Japan

## Abstract

**Background:**

The Japanese Gastric Cancer Association (JGCA) started a new nationwide gastric cancer registration in 2008.

**Methods:**

From 208 participating hospitals, 53 items including surgical procedures, pathological diagnosis, and survival outcomes of 13,626 patients with primary gastric cancer treated in 2002 were collected retrospectively. Data were entered into the JGCA database according to the JGCA classification (13th edition) and UICC TNM classification (5th edition) using an electronic data collecting system. Finally, data of 13,002 patients who underwent laparotomy were analyzed.

**Results:**

The 5-year follow-up rate was 83.3 %. The direct death rate was 0.48 %. UICC 5-year survival rates (5YEARSs)/JGCA 5YEARSs were 92.2 %/92.3 % for stage IA, 85.3 %/84.7 % for stage IB, 72.1 %/70.0 % for stage II, 52.8 %/46.8 % for stage IIIA, 31.0 %/28.8 % for stage IIIB, and 14.9 %/15.3 % for stage IV, respectively. The proportion of patients more than 80 years old was 7.8 %, and their 5YEARS was 51.6 %. Postoperative outcome of the patients with primary gastric carcinoma in Japan have apparently improved in advanced cases and among the aged population when compared with the archival data. Further efforts to improve the follow-up rate are needed.

**Conclusions:**

Postoperative outcome of the patients with primary gastric carcinoma in Japan have apparently improved in advanced cases and among the aged population when compared with the archival data. Further efforts to improve the follow-up rate are needed.

## Introduction

The registration committee of the Japanese Gastric Cancer Association (JGCA) started a new registration program in 2008 after a 10-year blank period, and we reported the 5-year follow-up data of the patients treated in 2001 [1]. The registration has been continuing, and here we report the results of those treated in 2002.

## Materials and methods

Leading hospitals in Japan voluntarily downloaded and fulfilled the database provided by the JGCA and sent the anonymized data to the JGCA data center. The collected data were analyzed according to the previously reported methods [1].

## Results

**Fig. 1:**
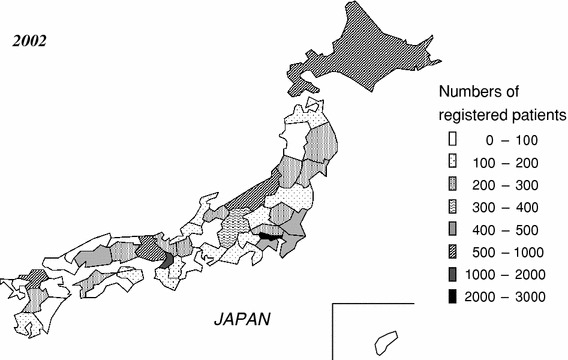
Geographic distribution of registered patients by prefecture

Data of 14,394 patients were collected from 208 hospitals; 126 (60.6 %) hospitals participated in both years, but 82 hospitals were new, which was a 10 % increase as compared to the previous year (13,067 patients from 187 hospitals). The geographic distribution of the registered patients among the 47 prefectures is illustrated in Fig. 1. In Tokyo, 2,332 patients per year were registered, followed by 1,464 in Osaka. Four other prefectures registered more than 500 patients. On the other hand, the number of registered patients was fewer than 100 in 10 prefectures, and there were no registered patients in 2 prefectures.

Patients with remnant stomach cancer, non-epithelial malignant tumor, and gastric cancer combined with malignant tumor of other organs were excluded. Patients who were treated by endoscopic mucosal resection were also excluded. Data of 768 patients lacked essential items. Consequently, data of the remaining 13,002 patients were used for the final analysis.

**Fig. 2:**
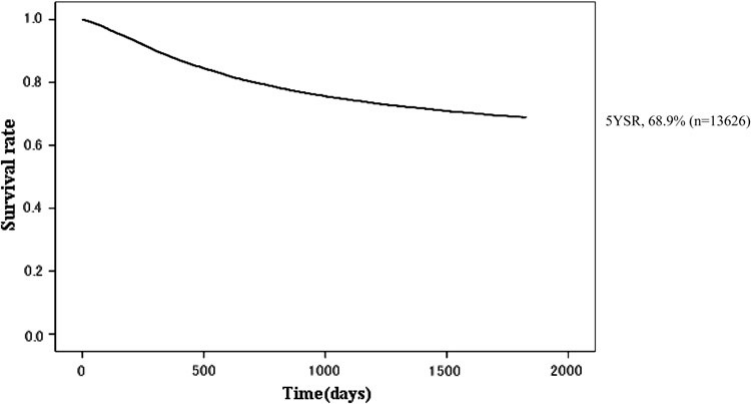
Kaplan–Meier survival for all patients with primary gastric cancer. *5YEARS* 5-year survival rate

**Fig. 3:**
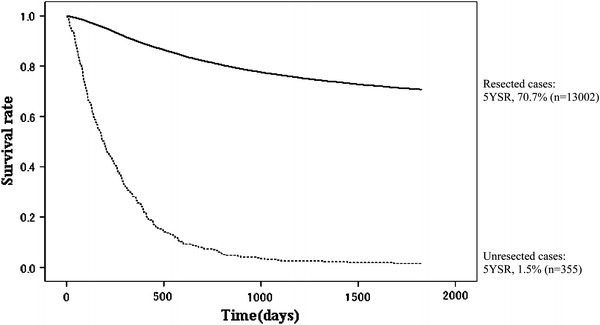
Kaplan–Meier survival for resected cases and unresected cases

**Fig. 4:**
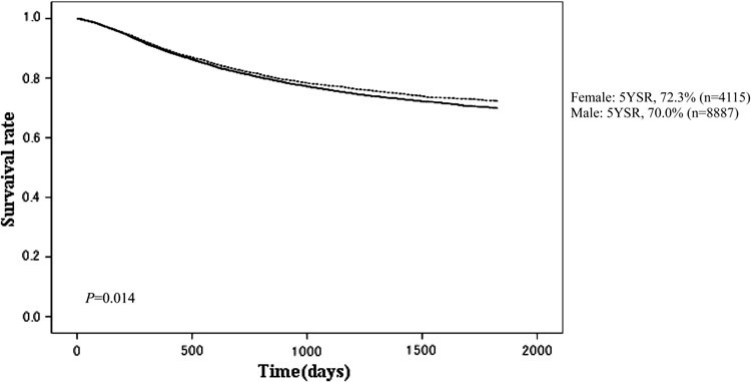
Kaplan–Meier survival of resected cases stratified by sex

**Fig. 5:**
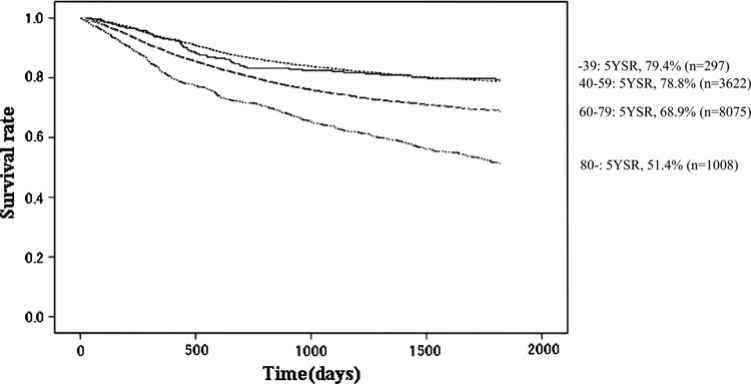
Kaplan–Meier survival of resected cases stratified by age

**Fig. 6:**
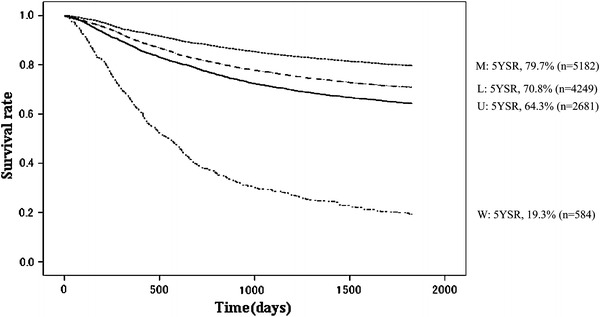
Kaplan–Meier survival of resected cases stratified by tumor location. *W* whole stomach

**Fig. 7:**
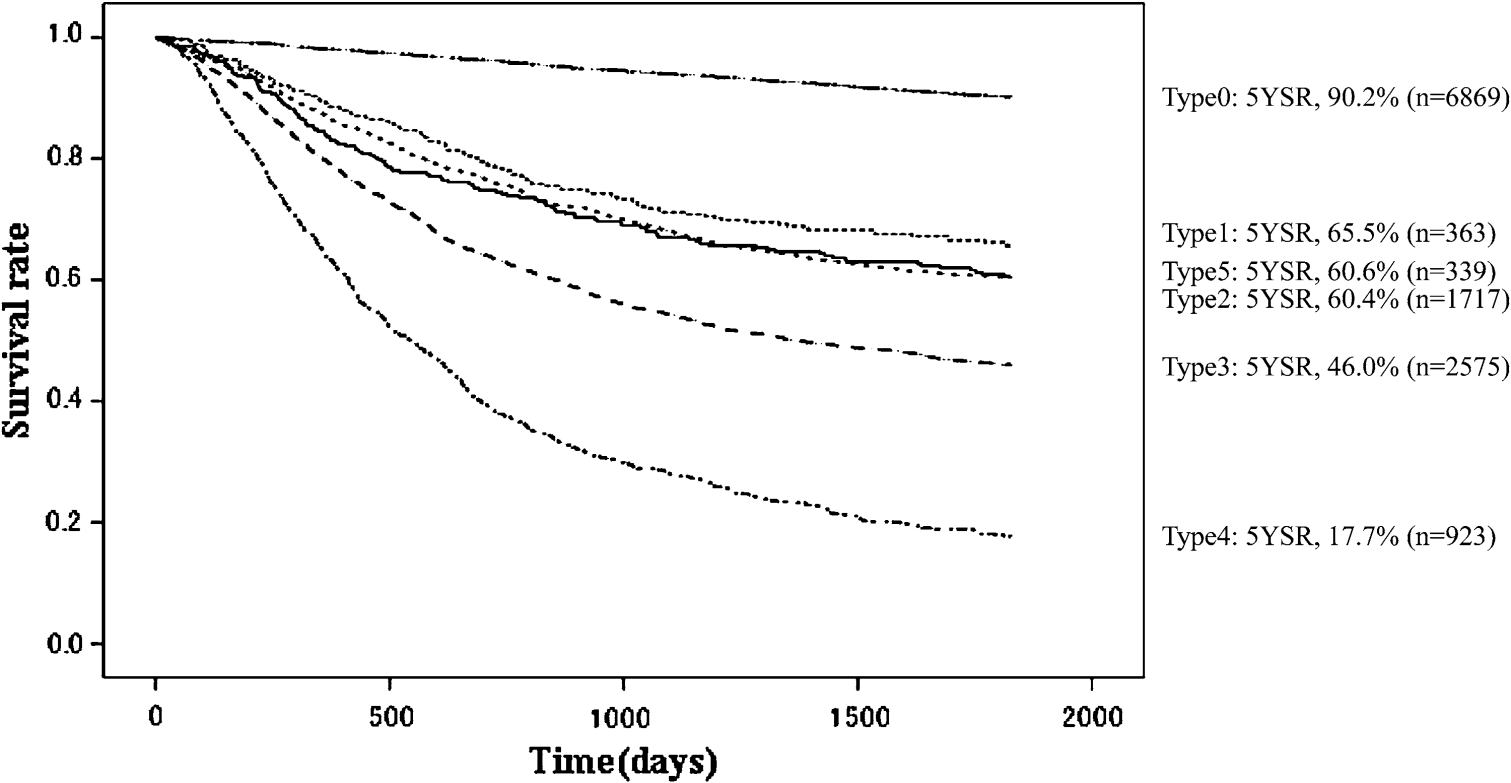
Kaplan–Meier survival of resected cases stratified by macroscopic type

**Fig. 8:**
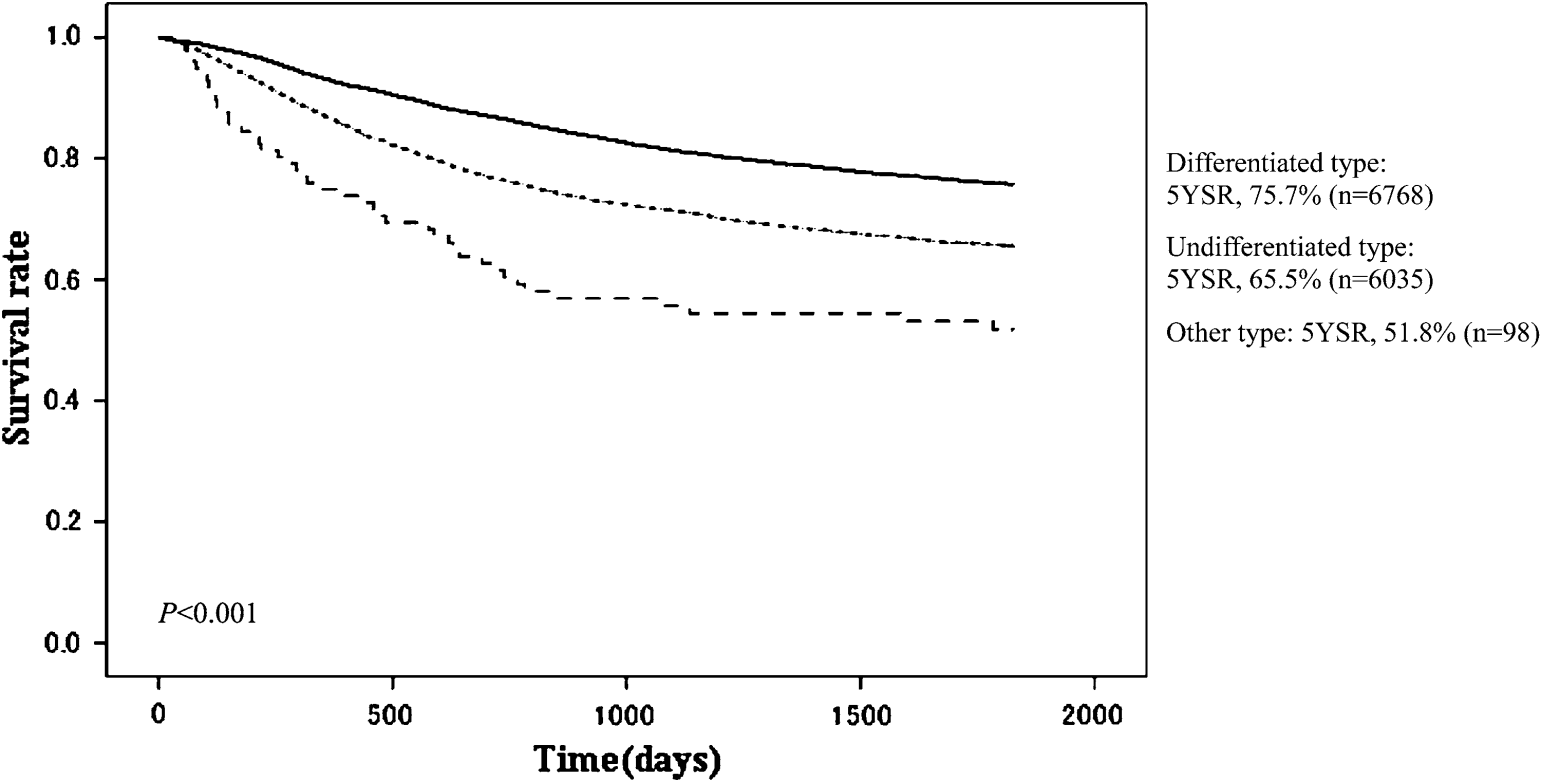
Kaplan–Meier survival of resected cases stratified by histological findings

**Fig. 9:**
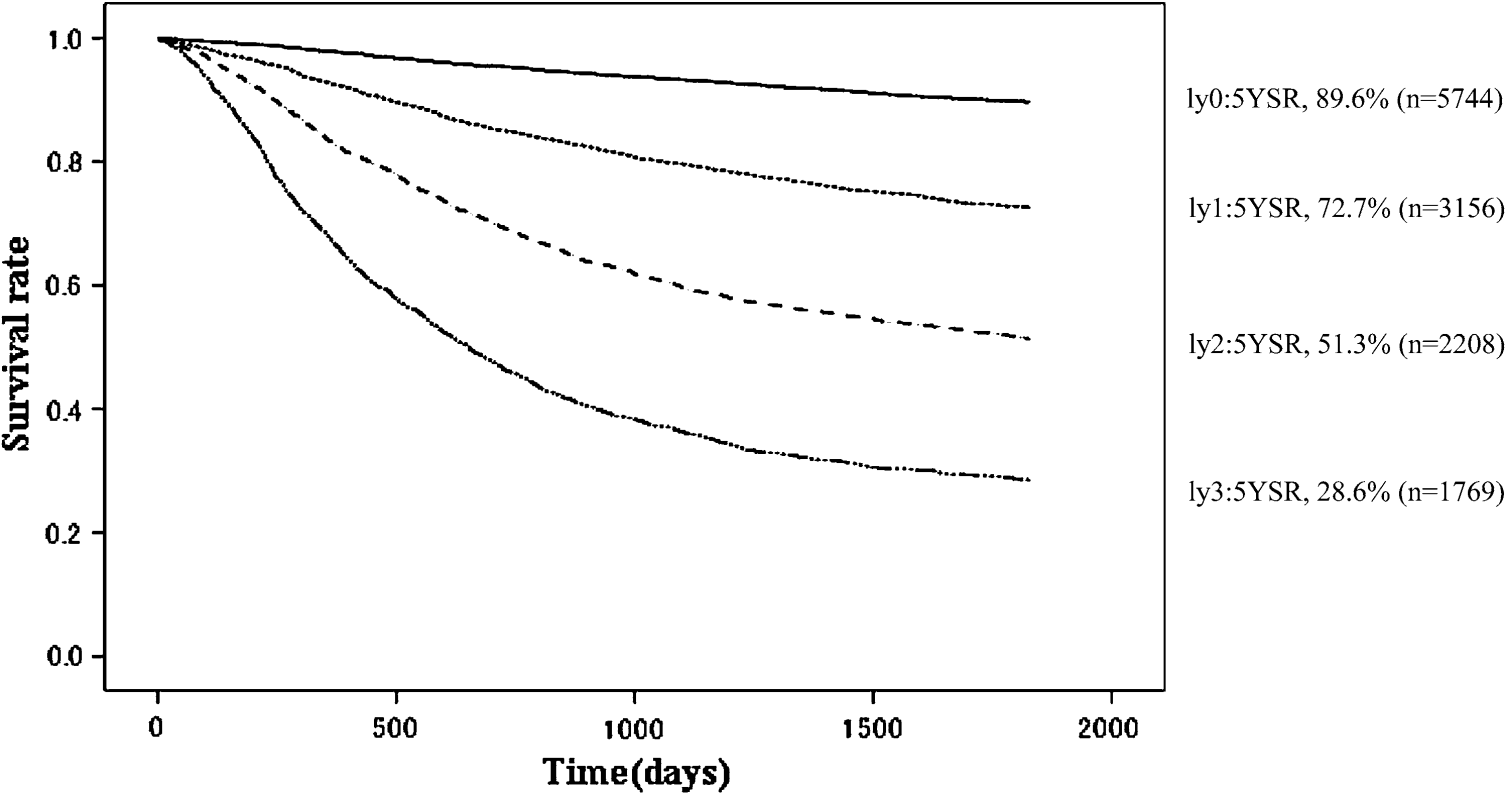
Kaplan–Meier survival of resected cases stratified by lymphatic invasion

**Fig. 10:**
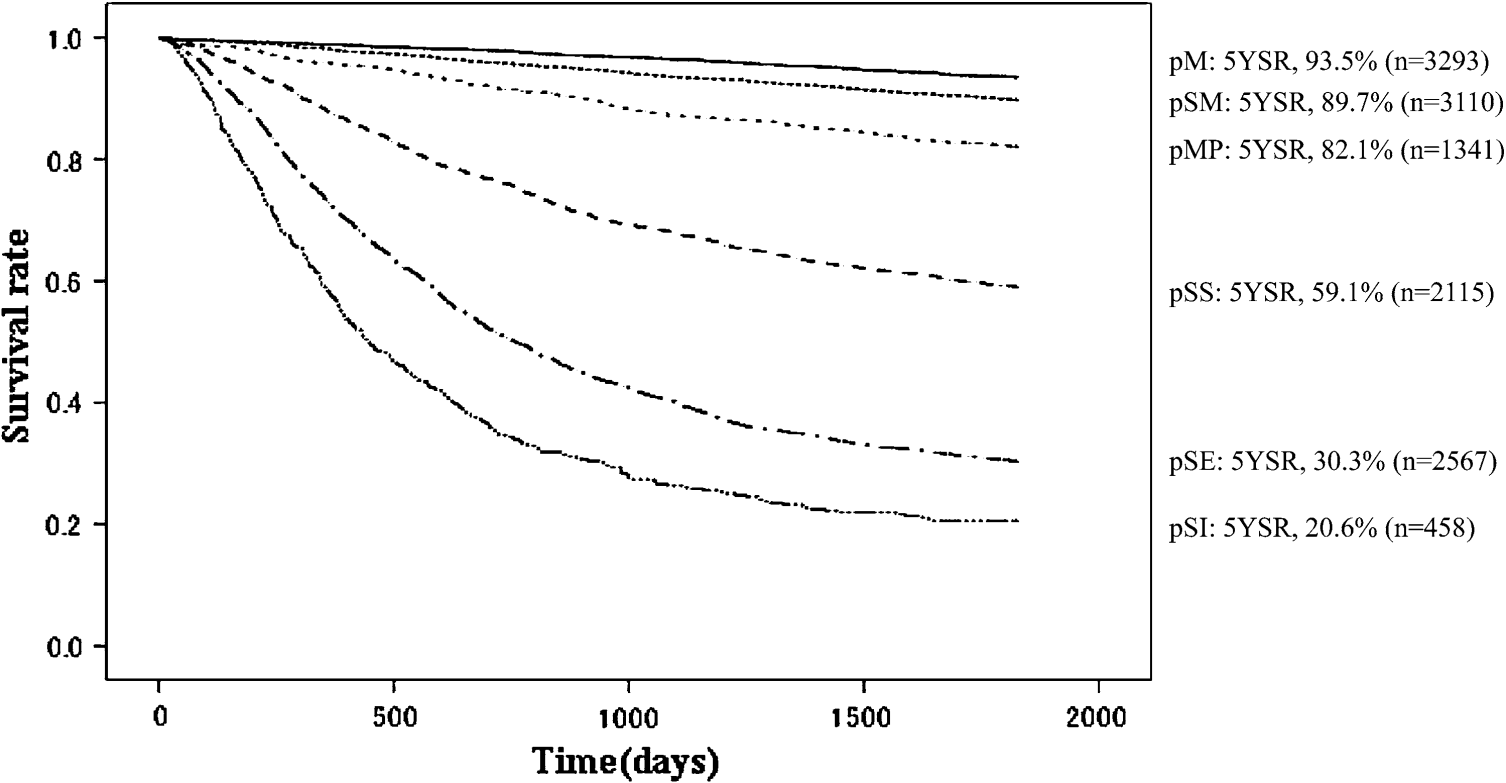
Kaplan–Meier survival of resected cases stratified by depth of tumor invasion

**Fig. 11:**
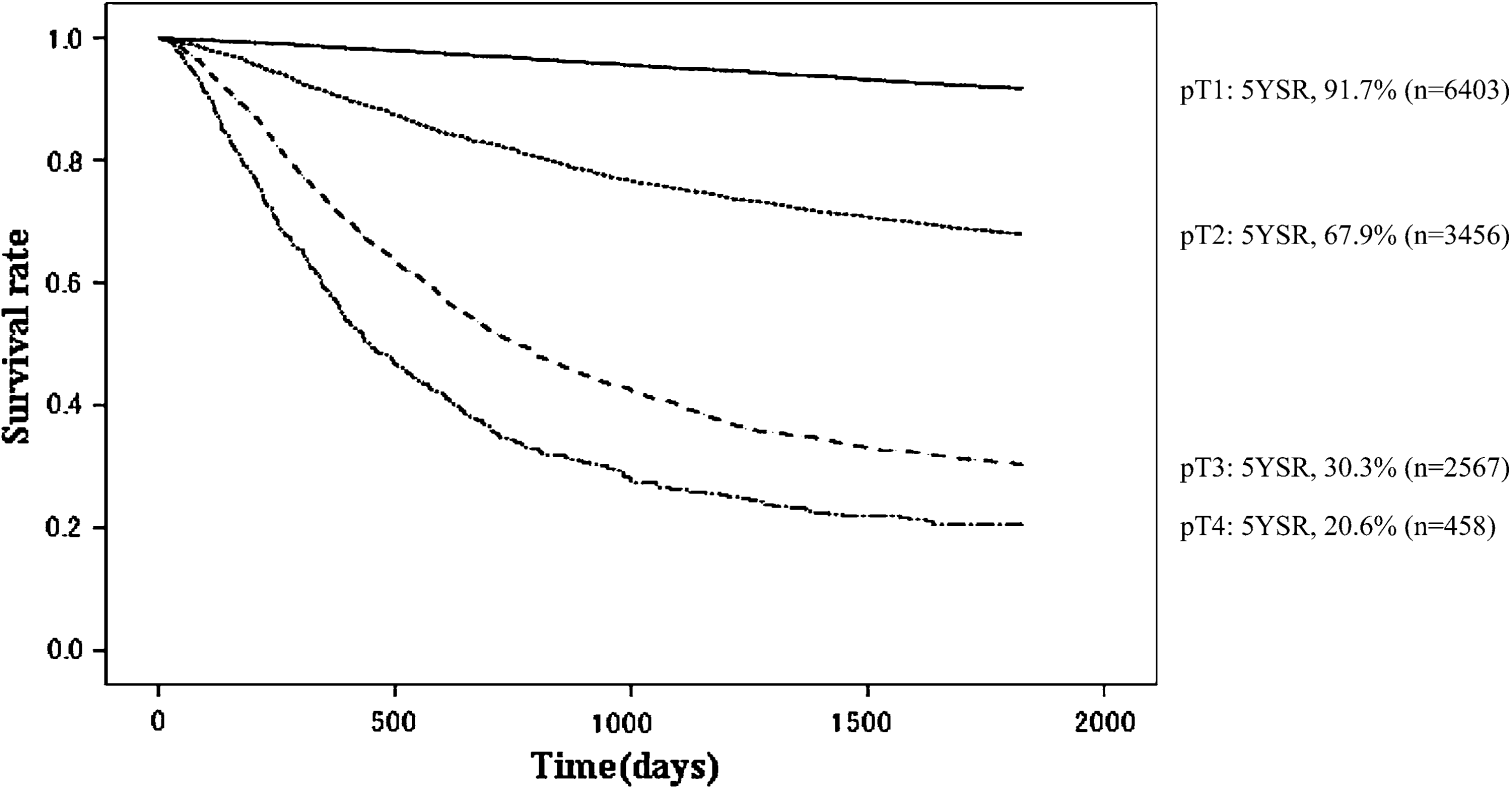
Kaplan–Meier survival of resected cases stratified by pT classification

**Fig. 12:**
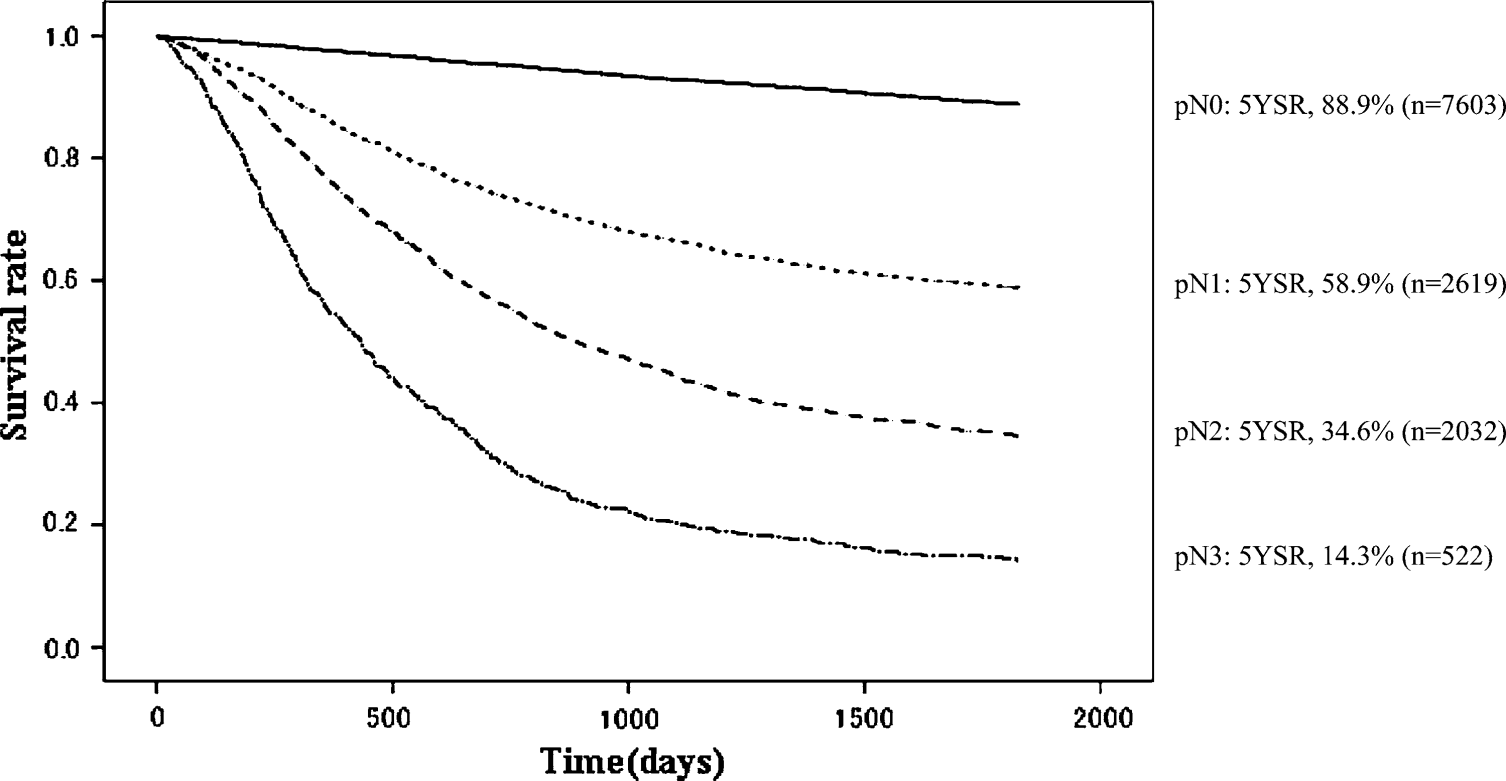
Kaplan–Meier survival of resected cases stratified by lymph node metastasis

**Fig. 13:**
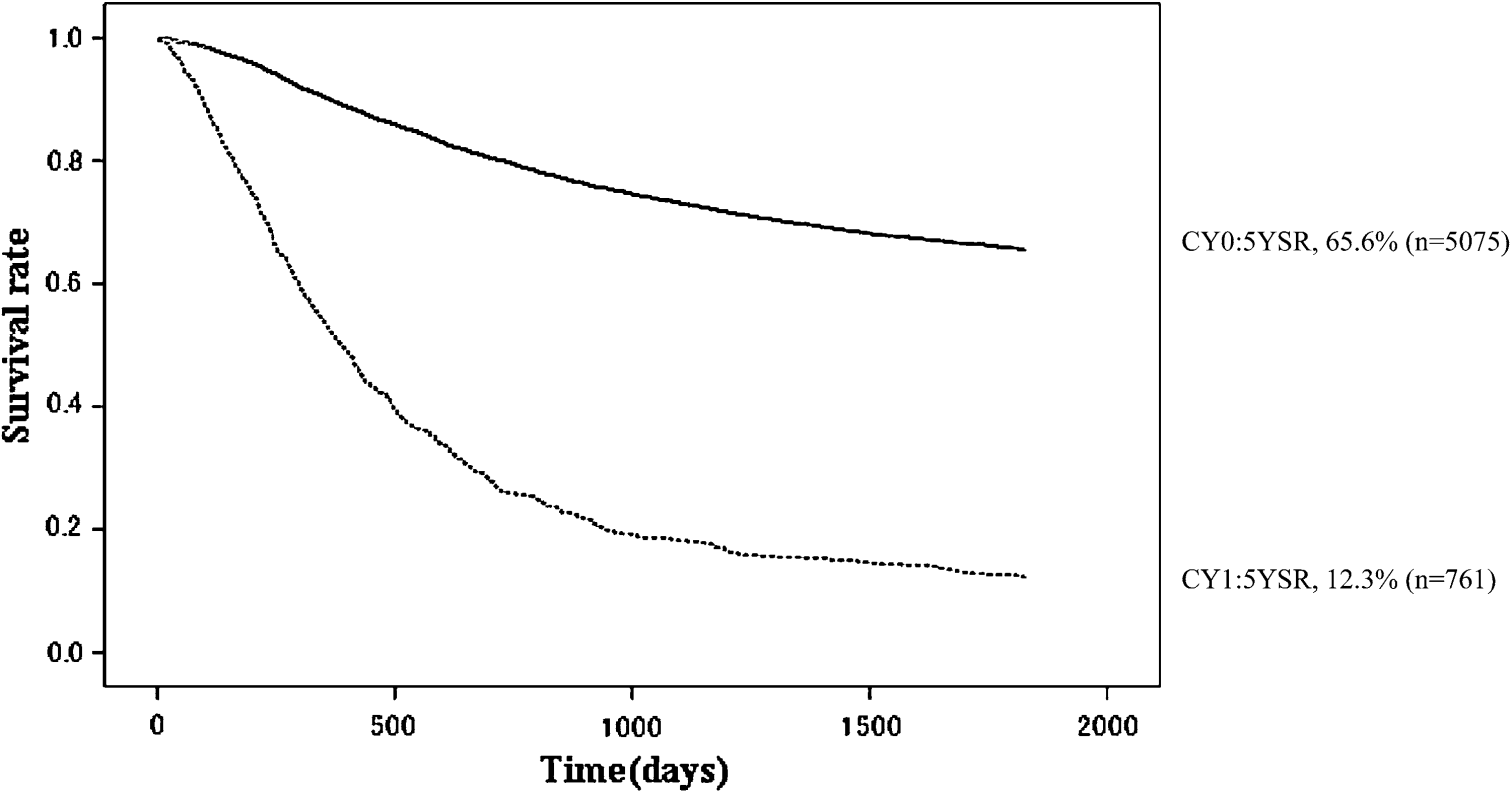
Kaplan–Meier survival of resected cases stratified by peritoneal cytology

**Fig. 14:**
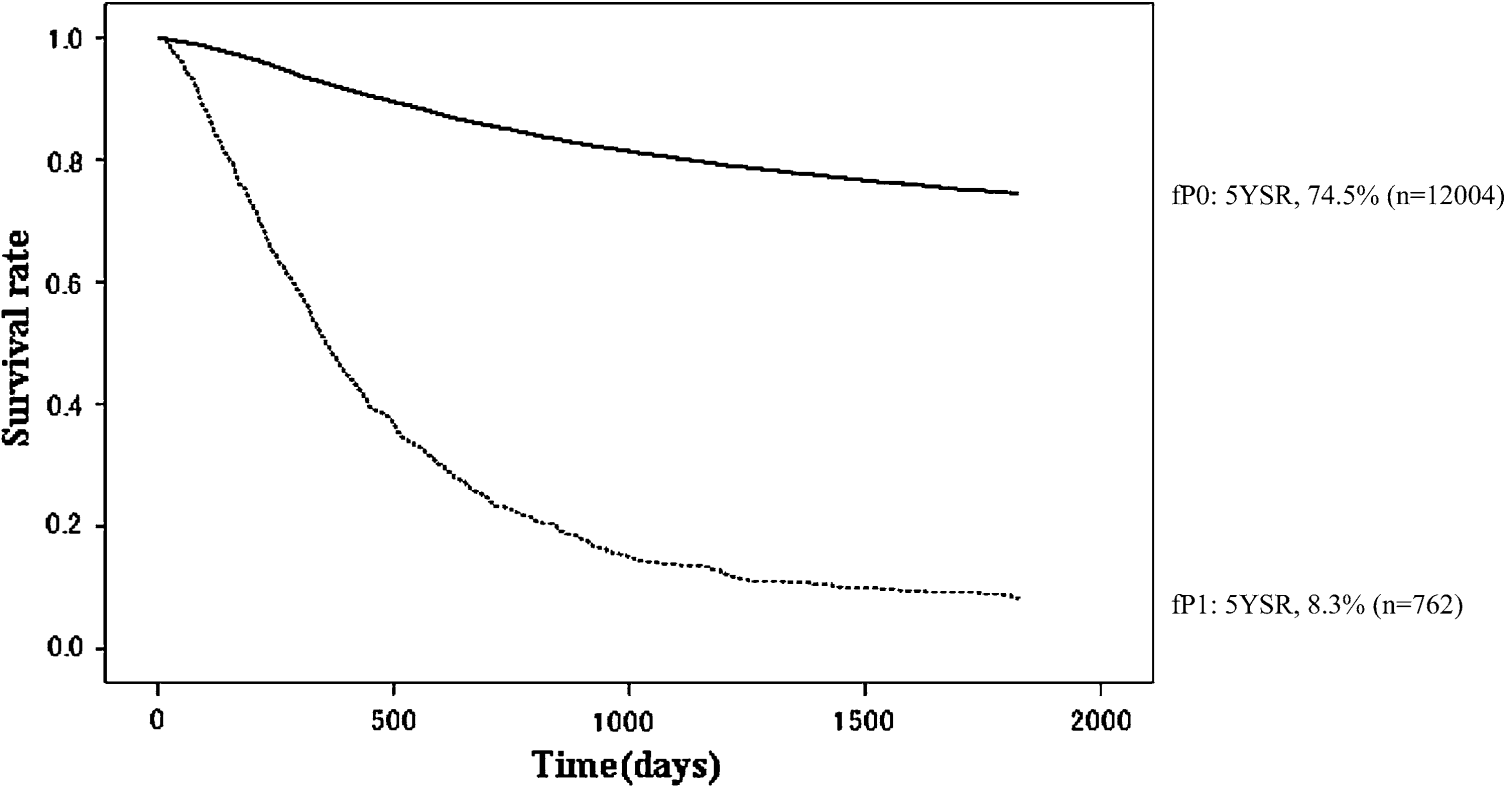
Kaplan–Meier survival of resected cases stratified by peritoneal metastasis

**Fig. 15:**
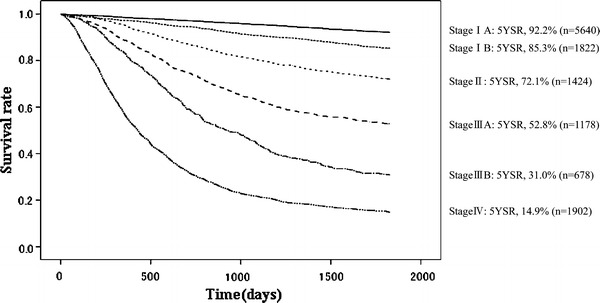
Kaplan–Meier survival of resected cases stratified by Japanese Gastric Cancer Association (JGCA) stage

**Fig. 16:**
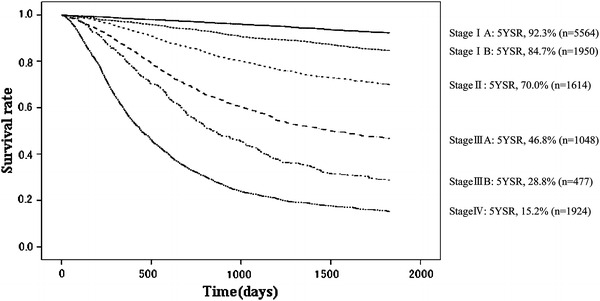
Kaplan–Meier survival of resected cases stratified by TNM stage

**Fig. 17:**
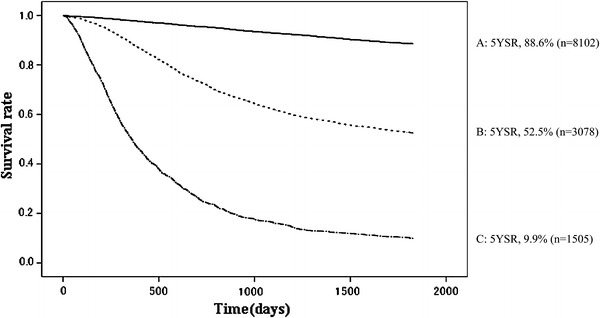
Kaplan–Meier survival of resected cases stratified by curative potential of gastric resection

The results are shown in Tables 1, 2, 3, 4, 5, 6, 7, 8, 9, 10, 11, 12, 13, 14, 15, 16, 17, 18, 19, 20, 21, 22, 23, 24, 25, 26, 27, and 28. Data given for each category of patients are: total number of patients, survival rates by year, standard error of 5YEARS, the number of direct death within 30 postoperative days, the number of patients lost to follow-up within 5 years, the number of 5-year survivors, and main cause of death, such as local and/or lymph node metastasis, peritoneal metastasis, liver metastasis, distant metastasis, recurrence at unknown site, other cancer, and other disease. Figures 2, 3, 4, 5, 6, 7, 8, 9, 10, 11, 12, 13, 14, 15, 16, and 17 provide cumulative survival curves of patients stratified by essential categories.

**Table 1:** Primary cancer

Categories	No. of patients	Direct death	Lost f.u.	1 years (%)	2 years (%)	3 years (%)	4 years (%)	5 years (%)	SE at 5 years	Alive	Local rec.	Peritoneal	Liver rec.	Distant meta.	*R*	Other cancer	Other disease	Unknown
Primary cancer	13626	89	2233	88.1	79.6	74.5	71.2	68.9	0.4	7436	454	1483	388	243	322	167	567	333

*lost f.u.* lost to follow-up, *years(%)* years of cumulative survival rate, *SE* standard error, *rec* recurrence, *peritoneal* peritoneal recurrence, *R* recurrence of unknown site

**Table 2:** Resected cases and unresected cases and other surgeries

Categories	No. of patients	Direct death	Lost f.u.	1 year (%)	2 years (%)	3 years (%)	4 years (%)	5 years (%)	SE at 5 years	Alive	Local rec.	Peritoneal	Liver rec.	Distant meta.	*R*	Other cancer	Other disease	Unknown
Resected cases	13002	63	2173	89.8	81.6	76.5	73.1	70.7	0.4	7286	410	1283	357	215	278	158	539	303
Unresected cases	355	21	25	25.7	7.3	2.9	1.9	1.5	0.7	4	37	183	24	24	32	2	12	12

**Table 3:** Sex (resected cases)

Categories	No. of patients	Direct death	Lost f.u.	1 years (%)	2 years (%)	3 years (%)	4 years (%)	5 years (%)	SE at 5 years	Alive	Local rec.	Peritoneal	Liver rec.	Distant meta.	*R*	Other cancer	Other disease	Unknown
Male	8887	43	1464	89.7	81.4	76.1	72.5	70.0	0.5	4939	292	805	280	136	203	133	425	210
Female	4115	20	709	90.1	82.2	77.4	74.3	72.3	0.7	2347	118	478	77	79	75	25	114	93

**Table 4:** Age (resected cases)

Categories	No. of patients	Direct death	Lost f.u.	1 year (%)	2 years (%)	3 years (%)	4 years (%)	5 years (%)	SE at 5 years	Alive	Local rec.	Peritoneal	Liver rec.	Distant meta.	*R*	Other cancer	Other disease	Unknown
<39	297	0	50	93.0	83.2	82.1	80.2	79.4	2.4	190	5	36	1	4	4	0	1	6
40–59	3622	10	581	93.4	86.7	83.2	80.3	78.8	0.7	2316	78	327	67	61	64	28	42	58
60–79	8075	40	1279	89.1	80.5	74.8	71.4	68.9	0.5	4450	282	798	255	142	180	110	387	192
>80	1008	13	263	81.6	71.6	63.9	57.0	51.4	1.8	330	45	122	34	8	30	20	109	47

**Table 5:** Tumor location (resected cases)

Categories	No. of patients	Direct death	Lost f.u.	1 year (%)	2 years (%)	3 years (%)	4 years (%)	5 years (%)	SE at 5 years	Alive	Local rec.	Peritoneal	Liver rec.	Distant meta.	*R*	Other cancer	Other disease	Unknown
U	2681	18	434	87.3	77.5	71.1	67.1	64.3	1.0	1356	104	267	99	76	68	39	150	88
M	5182	8	881	93.6	88.4	84.4	81.7	79.7	0.6	3322	102	339	101	62	72	48	153	102
L	4249	28	766	90.3	81.8	76.8	73.2	70.8	0.7	2338	159	380	124	46	90	59	200	87
Whole	584	8	62	63.7	37.9	28.7	22.9	19.3	1.7	88	37	256	20	24	45	5	22	25

*U* upper third, *M* middle third, *L* lower third

**Table 6:** Macroscopic type (resected cases)

Categories	No. of patients	Direct death	Lost f.u.	1 year (%)	2 years (%)	3 years (%)	4 years (%)	5 years (%)	SE at 5 years	Alive	Local rec.	Peritoneal	Liver rec.	Distant meta.	*R*	Other cancer	Other disease	Unknown
Type0	6869	13	1294	98.1	96.1	94.0	92.1	90.2	0.4	4959	40	69	31	22	24	105	244	81
Type1	363	0	62	89.1	78.6	71.1	68.2	65.5	2.6	187	12	22	24	9	9	5	20	13
Type2	1717	21	291	87.0	75.8	68.1	63.0	60.4	1.2	798	86	147	118	49	61	20	105	42
Type3	2575	17	364	79.6	63.3	54.3	49.1	46.0	1.0	914	181	532	158	79	102	22	115	108
Type4	923	9	86	63.7	37.9	28.2	21.5	17.7	1.3	127	55	450	12	39	72	2	36	44
Type5	339	2	43	83.9	74.5	67.0	63.7	60.6	2.8	171	16	51	9	12	8	3	13	13

**Table 7:** Histological type (resected cases)

Categories	No. of patients	Direct death	Lost f.u.	1 years (%)	2 years (%)	3 years (%)	4 years (%)	5 years (%)	SE at 5 years	Alive	Local rec.	Peritoneal	Liver rec.	Distant meta.	*R*	Other cancer	Other disease	Unknown
Papillary adenocarcinoma (pap)	464	6	86	88.2	78.8	70.7	66.7	64.3	2.4	227	15	30	36	7	13	10	26	14
tub 1	2846	9	542	96.2	92.4	88.3	85.9	83.6	0.7	1877	32	71	47	14	29	51	146	37
tub 2	3458	18	585	91.0	82.8	77.0	73.3	70.8	0.8	1936	120	259	131	64	63	48	169	83
por 1	1746	10	301	85.0	75.1	70.1	66.3	64.4	1.2	867	81	192	72	41	63	13	69	47
por 2	2449	15	309	83.0	70.2	64.2	59.6	57.0	1.0	1148	120	530	43	63	80	16	81	59
Signet-ring cell carcinoma (sig)	1581	5	279	94.2	89.0	85.8	83.6	81.5	1.0	1030	18	127	4	17	18	16	30	42
Mucinous adenocarcinoma (muc)	259	0	34	83.7	68.6	61.9	59.3	55.2	3.2	116	14	53	4	5	6	2	11	14
Adenosquamous carcinoma	17	0	0	52.9	29.4	23.5	23.5	23.5	10.3	4	2	1	4	1	0	1	1	3
Squamous cell carcinoma	6	0	0	100.0	66.7	50.0	50.0	50.0	20.4	3	0	0	2	0	1	0	0	0
Miscellaneous carcinoma	75	0	18	77.9	69.0	64.2	62.4	58.8	6.1	29	4	6	11	1	1	0	2	3

*tub 1* tubular adenocarcinoma, well-differentiated type; *tub 2* tubular adenocarcinoma, moderately differentiated type; *por 1* poorly differentiated adenocarcinoma, solid type, *por 2*, poorly differentiated adenocarcinoma, non-solid type

**Table 8:** Histological findings (resected cases)

Categories	No. of patients	Direct death	Lost f.u.	1 year (%)	2 years (%)	3 years (%)	4 years (%)	5 years (%)	SE at 5 years	Alive	Local rec.	Peritoneal	Liver rec.	Distant meta.	*R*	Other cancer	Other disease	Unknown
Differentiated type	6768	33	1213	93.0	86.6	81.3	78.1	75.7	0.5	4040	167	360	214	85	105	109	341	134
Undifferentiated type	6035	30	923	86.5	76.5	71.5	67.8	65.5	0.6	3161	233	902	123	126	167	47	191	162
Other type	98	0	18	74.9	61.5	55.7	54.4	51.8	5.3	36	6	7	17	2	2	1	3	6

**Table 9:** Lymphatic invasion(ly) (resected cases)

Categories	No. of patients	Direct death	Lost f.u.	1 year (%)	2 years (%)	3 years (%)	4 years (%)	5 years (%)	SE at 5 years	Alive	Local rec.	Peritoneal	Liver rec.	Distant meta.	*R*	Other cancer	Other disease	Unknown
ly0	5744	10	1089	97.8	95.3	93.3	91.3	89.6	0.4	4108	22	108	21	21	23	80	202	70
ly1	3156	16	524	92.6	84.9	79.7	75.4	72.7	0.8	1833	67	278	88	42	58	38	148	80
ly2	2208	14	321	83.2	69.1	59.8	54.9	51.3	1.1	891	135	370	142	74	87	25	103	60
ly3	1769	23	217	67.1	46.1	36.4	31.2	28.6	1.1	387	183	516	105	77	103	12	78	91

**Table 10:** Venous invasion(v) (resected cases)

Categories	No. of patients	Direct death	Lost f.u.	1 years (%)	2 years (%)	3 years (%)	4 years (%)	5 years (%)	SE at 5 years	Alive	Local rec.	Peritoneal	Liver rec.	Distant meta.	*R*	Other cancer	Other disease	Unknown
v0	8027	22	1456	95.8	91.6	88.3	85.8	83.4	0.4	5344	105	384	68	57	69	107	308	129
v1	2800	21	405	85.1	72.9	65.1	60.3	57.6	1.0	1284	146	446	110	81	84	32	121	91
v2	1347	11	183	75.5	57.8	48.3	42.9	40.8	1.4	425	100	291	97	44	75	13	68	51
v3	676	9	104	66.1	45.5	38.2	33.3	31.5	1.9	151	54	145	80	31	44	3	34	30

**Table 11:** Depth of invasion (resected cases)

Categories	No. of patients	Direct death	Lost f.u.	1 year (%)	2 years (%)	3 years (%)	4 years (%)	5 years (%)	SE at 5 years	Alive	Local rec.	Peritoneal	Liver rec.	Distant meta.	*R*	Other cancer	Other disease	Unknown
pT1(M)	3293	9	689	98.8	97.8	96.4	94.9	93.5	0.5	2410	3	7	3	2	4	40	109	26
pT1(SM)	3110	6	550	98.0	95.8	93.5	91.7	89.7	0.6	2268	17	12	17	13	9	51	129	44
pT2(MP)	1341	4	252	95.8	91.5	87.2	84.8	82.1	1.1	869	25	31	27	16	13	17	62	29
pT2(SS)	2115	14	306	87.8	76.0	67.9	62.5	59.1	1.1	996	110	236	128	73	69	29	104	64
pT3(SE)	2567	26	301	72.5	51.0	40.3	33.6	30.3	1.0	614	192	839	153	94	138	14	109	113
pT4(SI)	458	4	52	57.7	34.6	26.3	21.9	20.6	2.0	68	47	154	28	17	42	4	21	25

**Table 12:** pT classification (resected cases)

Categories	No. of patients	Direct death	Lost f.u.	1 year (%)	2 years (%)	3 years (%)	4 years (%)	5 years (%)	SE at 5 years	Alive	Local rec.	Peritoneal	Liver rec.	Distant meta.	*R*	Other cancer	Other disease	Unknown
pT1	6403	15	1239	98.4	96.9	95.0	93.3	91.7	0.4	4678	20	19	20	15	13	91	238	70
pT2	3456	18	558	90.9	82.0	75.3	71.1	67.9	0.8	1865	135	267	155	89	82	46	166	93
pT3	2567	26	301	72.5	51.0	40.3	33.6	30.3	1.0	614	192	839	153	94	138	14	109	113
pT4	458	4	52	57.7	34.6	26.3	21.9	20.6	2.0	68	47	154	28	17	42	4	21	25

**Table 13:** Lymph node metastasis (resected cases)

categories	No. of patients	Direct death	Lost f.u.	1 yearr (%)	2 years (%)	3 years (%)	4 years (%)	5 years (%)	SE at 5 years	Alive	Local rec.	Peritoneal	Liver rec.	Distant meta.	*R*	Other cancer	Other disease	Unknown
pN0	7603	20	1482	97.6	95.3	92.9	90.9	88.9	0.4	5350	29	132	52	21	34	107	303	93
pN1	2619	17	374	86.3	73.9	66.6	61.4	58.9	1.0	1240	115	402	124	59	81	28	124	72
pN2	2032	15	246	76.4	56.0	44.5	38.1	34.6	1.1	547	172	542	132	88	114	15	79	97
pN3	522	9	41	54.9	30.1	20.3	16.5	14.3	1.6	61	86	158	36	43	41	3	22	31

**Table 14:** Peritoneal cytology (resected cases)

Categories	No. of patients	Direct death	Lost f.u.	1 year (%)	2 years (%)	3 years (%)	4 years (%)	5 years (%)	SE at 5 years	Alive	Local rec.	Peritoneal	Liver rec	Distant meta.	*R*	Other cancer	Other disease	Unknown
CY0	5075	16	761	89.9	80.0	73.2	68.6	65.6	0.7	2675	229	576	200	117	112	60	199	146
CY1	761	16	71	52.2	26.1	18.3	15.0	12.3	1.3	72	45	386	28	36	52	2	33	36

**Table 15:** Peritoneal metastasis (P) (resected cases)

Categories	No. of patients	Direct death	Lost f.u.	1 year(%)	2 years (%)	3 years (%)	4 years (%)	5 years (%)	SE at 5 years	Alive	Local rec.	Peritoneal	Liver rec.	Distant meta.	*R*	Other cancer	Other disease	Unknown
fP0	12004	47	2082	92.3	85.2	80.3	77.0	74.5	0.4	7087	349	862	308	184	218	154	503	257
fP1	762	15	62	48.9	23.3	13.9	9.9	8.3	1.1	49	48	402	44	28	56	4	31	38

**Table 16:** Liver metastasis (H) (resected cases)

Categories	No. of patients	Direct death	Lost f.u.	1 year (%)	2 years (%)	3 years (%)	4 years (%)	5 years (%)	SE at 5 years	Alive	Local rec.	Peritoneal	Liver rec.	Distant meta.	*R*	Other cancer	Other disease	Unknown
fH0	12441	57	2114	91.0	83.1	78.0	74.6	72.2	0.4	7114	386	1197	229	200	247	156	517	281
fH1	326	6	34	39.8	22.3	15.5	12.7	11.4	1.9	23	10	63	122	12	28	0	17	17

**Table 17:** Distant metastasis including peritoneal and liver metastasis (M) (resected cases)

Categories	No. of patients	Direct death	Lost f.u.	1 year (%)	2 years (%)	3 years (%)	4 years (%)	5 years (%)	SE at 5 years	Alive	Local rec.	Peritoneal	Liver rec	Distant meta.	*R*	Other cancer	Other disease	Unknown
fM0	12530	56	2128	90.4	82.5	77.5	74.1	71.7	0.4	7104	376	1186	322	185	262	155	518	294
fM1	216	6	15	53.2	29.5	18.1	13.5	12.4	2.4	22	21	73	28	26	15	1	11	4

**Table 18:** Japanese stage (resected cases)

Categories	No. of patients	Direct death	Lost f.u.	1 year (%)	2 years (%)	3 years (%)	4 years (%)	5 years (%)	SE at 5 years	Alive	Local rec.	Peritoneal	Liver rec.	Distant meta.	*R*	Other cancer	Other disease	Unknown
StageIA	5640	14	1113	98.5	97.1	95.4	93.8	92.2	0.4	4126	11	11	10	4	11	86	215	53
StageIB	1822	5	364	97.2	94.4	90.8	88.1	85.3	0.9	1216	14	41	25	15	12	27	79	29
StageII	1424	3	220	95.0	86.5	80.2	75.5	72.1	1.2	834	50	100	43	30	30	15	69	33
StageIIIA	1178	6	159	88.6	74.0	63.1	56.1	52.8	1.5	501	81	199	55	30	40	14	56	43
StageIIIB	678	4	85	82.1	58.0	43.8	34.9	31.0	1.9	161	61	205	38	31	31	5	32	29
StageIV	1902	30	180	55.6	31.0	21.7	17.4	14.9	0.9	218	180	708	180	101	149	8	80	98

**Table 19:** Japanese stage (resected cases)

Categories	No. of patients	Direct death	Lost f.u.	1 year (%)	2 years (%)	3 years (%)	4 years (%)	5 years (%)	SE at 5 years	Alive	Local rec.	Peritoneal	Liver rec.	Distant meta.	*R*	Other cancer	Other disease	Unknown
StageI	7462	19	1477	98.2	96.4	94.3	92.4	90.5	0.4	5342	25	52	35	19	23	113	294	82
StageII	1424	3	220	95.0	86.5	80.2	75.5	72.1	1.2	834	50	100	43	30	30	15	69	33
StageIII	1856	10	244	86.2	68.2	56.1	48.4	44.9	1.2	662	142	404	93	61	71	19	88	72
StageIV	1902	30	180	55.6	31.0	21.7	17.4	14.9	0.9	218	180	708	180	101	149	8	80	98

**Table 20:** TNM stage (resected cases)

Categories	No. of patients	Direct death	Lost f.u.	1 year (%)	2 years (%)	3 years (%)	4 years (%)	5 years (%)	SE at 5 years	Alive	Local rec.	Peritoneal	Liver rec.	Distant meta.	*R*	Other cancer	Other disease	Unknown
Stage IA	5564	15	1111	98.2	97.1	95.5	93.9	92.3	0.4	4062	10	9	9	4	11	84	210	54
Stage IB	1950	5	385	97.0	93.8	89.9	87.5	84.7	0.9	1294	17	49	25	15	20	28	84	33
Stage II	1614	5	261	94.0	85.4	78.4	73.3	70.0	1.2	903	62	125	64	35	34	14	80	36
Stage IIIA	1048	9	133	86.1	68.4	58.2	50.6	46.8	1.6	399	75	204	44	33	37	15	55	53
Stage IIIB	477	1	58	79.6	55.6	41.9	32.2	28.8	2.2	107	45	166	19	18	28	4	17	15
Stage IV	1924	27	184	57.3	32.8	22.4	17.9	15.2	0.9	223	180	704	189	107	142	12	83	100

**Table 21:** TNM stage (resected cases)

Categories	No. of patients	Direct death	Lost f.u.	1 year (%)	2 years (%)	3 years (%)	4 years (%)	5 years (%)	SE at 5 years	Alive	Local rec.	Peritoneal	Liver rec.	Distant meta.	*R*	Other cancer	Other disease	Unknown
StageI	7514	20	1496	98.1	96.2	94.1	92.2	90.3	0.4	5356	27	58	34	19	31	112	294	87
StageII	1614	5	261	94.0	85.4	78.4	73.3	70.0	1.2	903	62	125	64	35	34	14	80	36
StageIII	1525	10	191	84.1	64.4	53.2	44.9	41.2	1.3	506	120	370	63	51	65	19	72	68
StageIV	1924	27	184	57.3	32.8	22.4	17.9	15.2	0.9	223	180	704	189	107	142	12	83	100

**Table 22:** Surgical approach (resected cases)

Categories	No. of patients	Direct death	Lost f.u.	1 year (%)	2 years (%)	3 years (%)	4 years (%)	5 years (%)	SE at 5 years	Alive	Local rec.	Peritoneal	Liver rec.	Distant meta.	*R*	Other cancer	Other disease	Unknown
Laparotomy	12166	59	2021	89.6	81.2	76.0	72.4	69.9	0.4	6745	391	1238	346	204	273	147	514	287
Thoracolaparotomy	152	2	13	70.7	52.0	41.4	38.5	35.4	4.0	45	14	35	6	10	5	3	12	9
Laparoscopic	658	2	136	97.7	96.4	95.0	94.4	93.3	1.0	481	4	6	4	0	0	8	13	6
Others	6	0	2	80.0	60.0	60.0	60.0	60.0	21.9	2	1	0	0	0	0	0	0	1

**Table 23:** Surgical procedures (resected cases)

Categories	No. of patients	Direct death	Lost f.u.	1 year (%)	2 years (%)	3 years (%)	4 years (%)	5 years (%)	SE at 5 years	Alive	Local rec.	Peritoneal	Liver rec.	Distant meta.	*R*	Other cancer	Other disease	Unknown
Distal gastrectomy	7743	32	1405	93.1	86.9	82.6	79.9	77.7	0.5	4742	197	515	179	72	124	83	283	143
Total gastrectomy	3966	25	548	81.2	67.5	60.1	54.9	51.9	0.8	1635	207	752	164	138	145	45	194	138
Proximal gastrectomy	523	2	111	94.8	91.3	88.3	86.5	85.1	1.6	341	5	12	9	2	4	7	22	10
Pylorus-preserving gastrectomy	397	1	37	99.5	98.2	95.9	94.8	92.6	1.3	332	1	2	3	1	1	3	14	3
Segmental or local gastrectomy	351	3	67	95.0	91.2	86.2	82.9	81.2	2.2	224	0	2	2	2	4	17	24	9
Surgical mucosal resection	22	0	5	100.0	89.5	78.9	78.9	73.3	10.2	12	0	0	0	0	0	3	2	0

**Table 24:** Lymph node dissection (D) (resected cases)

Categories	No. of patients	Direct death	Lost f.u.	1 ysr (%)	2 ysr (%)	3 ysr (%)	4 ysr (%)	5 ysr (%)	SE at 5 ysr	Alive	Local rec.	Peri-toneal	Liver rec.	Distant meta.	*R*	Other cancer	Other disease	Un-known
D0	802	12	125	80.5	73.7	69.1	67.0	65.6	1.7	420	17	95	34	10	17	18	49	17
D1	2553	15	457	86.4	79.1	74.6	71.2	68.8	1.0	1356	58	276	65	29	56	48	145	63
D1 + α	1684	7	349	92.0	86.1	83.2	80.9	78.6	1.1	1008	39	94	27	13	31	21	77	25
D1 + β	882	2	165	93.5	88.3	85.6	83.5	81.4	1.4	563	18	45	19	8	9	9	33	13
D2	6056	20	907	91.6	82.2	76.0	72.1	69.6	0.6	3424	240	654	183	126	124	53	201	144
D3	343	2	35	82.8	66.6	58.4	51.1	47.7	2.8	138	28	67	21	17	17	3	6	11

**Table 25:** Resection margins (resected cases)

Categories	No. of patients	Direct death	Lost f.u.	1 year (%)	2 years (%)	3 years (%)	4 years (%)	5 years (%)	SE at 5 years	Alive	Local rec.	Peritoneal	Liver rec.	Distant meta.	*R*	Other cancer	Other disease	Unknown
PM− and DM−	12217	56	2089	91.0	83.1	78.1	74.7	72.3	0.4	6984	355	1102	332	192	240	155	500	268
PM+ and/or DM+	397	7	43	50.9	32.2	23.4	18.4	16.2	2.0	50	34	144	18	15	34	2	27	30

**Table 26:** Combined resection of neighboring organs (resected cases)

Categories	No. of patients	Direct death	Lost f.u.	1 year (%)	2 years (%)	3 years (%)	4 years (%)	5 years (%)	SE at 5 years	Alive	Local rec.	Peritoneal	Liver rec.	Distant meta.	*R*	Other cancer	Other disease	Unknown
No combined resection	7955	33	1494	92.0	85.6	81.4	78.6	76.5	0.5	4729	193	588	161	80	132	98	326	154
Combined resection	4309	29	615	85.1	73.2	66.3	61.5	58.7	0.8	2032	191	651	183	123	135	55	192	132

*PM* proximal margin, *DM* distal margin

**Table 27:** Combined resected organs (resected cases)

Categories	No. of patients	Direct death	Lost f.u.	1 year (%)	2 years (%)	3 years (%)	4 years (%)	5 years (%)	SE at 5 years	Alive	Local rec.	Peritoneal	Liver rec.	Distant meta.	*R*	Other cancer	Other disease	Unknown
Caudal pancreas	313	1	35	74.2	54.5	45.5	39.7	37.5	2.9	96	28	77	15	17	22	3	10	10
Spleen	1444	10	189	84.7	68.8	59.6	53.7	49.7	1.4	573	80	288	61	70	49	15	68	51
Transverse colon	101	1	19	71.6	52.3	43.0	38.0	36.7	5.2	25	5	27	4	2	9	0	7	3
Transverse mesocolon	53	1	7	82.9	61.4	53.2	40.3	38.1	7.0	15	3	15	2	1	4	0	5	1
Diaphragma	9	0	1	50.8	25.4	0.0	0.0	0.0	0.0	0	0	0	4	2	0	0	1	1
Liver	96	2	11	63.6	49.2	40.2	34.5	33.2	5.0	24	7	11	17	4	8	2	6	6
Gallbladder	2121	12	339	89.1	81.9	77.3	73.5	71.2	1.0	1215	59	213	73	22	40	28	80	52
Adrenal gland	10	0	0	90.0	80.0	80.0	80.0	80.0	12.6	8	1	0	0	0	0	0	0	1
Kidney	7	0	2	85.7	85.7	85.7	68.6	68.6	18.6	3	0	0	0	0	0	1	1	0
Small intestine	10	0	1	90.0	70.0	60.0	60.0	60.0	15.5	5	0	1	0	0	0	0	2	1
Abdominal wall	1	0	0	0.0	0.0	0.0	0.0	0.0	0.0	0	0	0	1	0	0	0	0	0
Ovary	22	0	3	67.4	52.1	41.7	41.7	41.7	11.0	7	0	11	0	1	0	0	0	0
Pancreas head (PD)	20	2	2	85.0	69.1	58.4	41.8	35.9	11.3	6	2	0	1	0	2	1	5	1
Others	66	0	4	86.4	75.6	67.9	67.9	67.9	5.8	41	1	5	3	2	1	4	2	3

*PD* pancreatoduodenectomy

**Table 28:** Curative potential (resected cases)

Categories	No. of patients	Direct death	Lost f.u.	1 year (%)	2 years (%)	3 years (%)	4 years (%)	5 years (%)	SE at 5 years	Alive	Local rec.	Peritoneal	Liver rec.	Distant meta.	*R*	Other cancer	Other disease	Unknown
A	8102	20	1585	97.8	95.4	92.9	90.6	88.6	0.4	5674	58	119	66	39	39	113	300	109
B	3078	14	398	88.3	72.5	62.1	56.1	52.5	0.9	1318	206	508	137	94	115	39	155	108
C	1505	28	149	49.0	24.7	16.4	12.1	9.9	0.8	109	126	624	150	78	120	3	71	75

The 5YEARS in 13,626 patients with primary gastric cancer was 68.9 % (Table 1; Fig. 2). During the 5-year follow-up, 2,233 patients were lost; the follow-up rate was 83.6 %. Of the 13,626 patients, 13,002 underwent gastric resection. Accordingly, the resection rate was 95.4 %, and the 5YEARS of the resected patients was 70.7 % (Table 2; Fig. 3). Sixty-three of 13,002 resected cases died within 30 days postoperatively. The direct death rate was 0.48 %. The frequent causes of death in patients who had undergone gastrectomy were peritoneal metastasis (*n* = 1,283), followed by other diseases (*n* = 539), local recurrence including node metastasis (*n* = 410), liver metastasis (*n* = 357), recurrence at unknown site (*n* = 278), and other cancer (*n* = 158).

The proportion of male patients was 68.4 % with 5YEARS of 70.0 %; for female patients 5YEARS was 72.3 %, which was better statistically (Table 3; Fig. 4). Patients more than 80 years old were 7.8 % of all patients, and their 5YEARS was 51.4 % (Table 4; Fig. 5). On the other hand, 5YEARS of the patients under 39 years old was 79.4 % (*P* < 0.001). Cancer was located in the upper-third of the stomach in 21.1 % of the cases, and its 5YEARS was relatively low at 64.3 % (Table 5; Fig. 6). Patients with type 4 cancer amounted to 7.2 %, and their 5YEARS was markedly low at 17.7 % (Table 6; Fig. 7). The 5YEARS of type 3 was 46.0 % and that of type 2 was 60.4 %. For histological type, frequency of the undifferentiated type including poorly differentiated adenocarcinoma, signet-ring cell carcinoma, and mucinous adenocarcinoma was 46.8 % and its 5YEARS was 65.5 %, which was inferior to that of the differentiated type (75.7 %, *P* < 0.001; Tables 7, 8; Fig. 8). The grade of lymphatic invasion (ly0–ly3) and venous invasion (v0–v3) showed significant correlations with the prognosis (Tables 9, 10; Fig. 9).

A high incidence of early-stage cancer remained characteristic in 2002, as shown in Tables 11 and 12. The proportion of pathological M and SM (pT1) cancer was 49.7 %, and its primary cause of death was not cancer recurrence (17.9 %, *n* = 87) or other cancer (18.7 %), but other diseases (49.0 %, *n* = 238). The proportion of pathological MP and SS (pT2) was 26.8 %, SE (pT3) 19.9 %, and SI (pT4) 3.6 %. The 5YEARS of these subsets were 67.9 %, 30.3 %, and 20.6 %, respectively (Figs. 10, 11). The primary cause of death in advanced cancer was cancer recurrence, and the peritoneal recurrence rate was remarkably high in the pT3 and pT4 subsets. For the lymph node metastasis, the proportion of pN0 was 59.5 %, pN1 20.4 %, pN2 15.9 %, and pN3 4.1 %, and the 5YEARS of each subset was 88.9 %, 58.9 %, 34.6 %, and 14.3 %, respectively (Table 13; Fig. 12).

Peritoneal washing cytology was carried out in 5,836 patients with advanced gastric cancer; the positive rate was 13.0 %. The 5YEARS of cytology-positive (CY1) patients was 12.3 %, which was almost as dismal as the 5YEARS of the P1 patients (8.3 %; Tables 14, 15; Figs. 13, 14). The 5YEARS of patients with liver metastasis (H1) was 11.4 %, and of those with other types of distant metastasis was 12.4 % (Tables 16, 17).

The 5YEARS of the patients stratified by JGCA staging system was 92.2 % for stage IA, 85.3 % for stage IB, 72.1 % for stage II, 52.8 % for stage IIIA, 31.0 % for stage IIIB, and 14.9 % for stage IV. These JGCA 5YEARSs seemed to correlate well with TNM 5YEARSs, which were 92.3 % for stage IA, 84.7 % for stage IB, 70.0 % for stage II, 46.8 % for stage IIIA, 28.8 % for stage IIIB, and 15.2 % for stage IV (Table 18, 19, 20, 21; Figs. 15, 16).

For operative procedures, the proportion of patients who underwent laparoscopic gastrectomy was only 5.1 % in 2002, and their 5YEARS was 93.3 % (Table 22). Eligibility for laparoscopic surgery was strictly limited at that time, and the laparoscopic approach was selected almost exclusively in patients with the preoperative diagnosis of early gastric cancer. Only 1.2 % of the patients were treated by thoracolaparotomy, and their 5YEARS was 35.4 %. Thoracolaparotomy was usually carried out in patients with advanced gastric cancer with esophageal invasion more than 3 cm in length. Total gastrectomy was performed for 30.5 % of the patients, and their 5YEARS was 51.9 % (Table 23). D2 lymph node dissection, a standard procedure for resectable advanced gastric cancer according to the JGCA treatment guidelines, was performed in 49.2 % of the patients (Table 24) [2, 3]. The risk of direct death among those who underwent D2 gastrectomy was only 0.3 %. The proportion of patients treated with less invasive surgery such as proximal gastrectomy, pylorus-preserving gastrectomy, segmental gastrectomy, and local resection of the stomach was 9.8 %. D0, D1, D1 + α, and D1 + β dissection were carried out in 6.5 %, 20.7 %, 13.7 %, and 7.2 % of the patients, respectively. D0 and D1 dissection were carried out mainly in patients with noncurative factors or poor surgical risks. The incidence of positive resection margin (PM+ and/or DM+) was 3.1 % (Table 25). Combined resection of other organs was performed in 35.1 % (Table 26). The frequent combined resected organs in patients who underwent gastrectomy were gallbladder (*n* = 2121), spleen (*n* = 1444), caudal pancreas (*n* = 313), transverse colon (*n* = 101), liver (*n* = 96), and so on in descending order (Table 27).

The curative potential of gastric resection was an important prognostic factor. The proportion of patients with no residual tumors with high probability of cure (resection A) was 63.9 %, and their 5YEARS was 88.6 %. On the other hand, patients with definite residual tumors (resection C) amounted to 11.9 % of all patients who underwent laparotomy, and their 5YEARS was 9.9 % (Table 28; Fig. 17).

## Discussion

Estimates of the worldwide incidence, mortality, and prevalence of 26 cancers in the year 2002 were available in the GLOBOCAN series of the International Agency for Research on Cancer [4]. With an estimated 934,000 new cases per year in 2002 (8.6 % of new cancer cases), the incidence of stomach cancer is in fourth place, after cancers of the lung, breast, and colon and rectum. It is the second most common cause of death from cancer (700,000 deaths annually).

The data presented in this report were collected from 208 hospitals in Japan. Cancer incidence rate (annual number of newly diagnosed cases per 100,000 population) in Japan in 2002 was approximately 520 for males and 370 for females. The incidences of various cancers in Japan are estimated from data collected by the cancer registry system in a dozen prefectures. According to these statistics, the number of cancer incidences in 2002 was approximately 589,000. The stomach was the leading site (21 %) for males and the second highest site (14 %) for females. The number of new patients who were diagnosed as gastric cancer in 2002 was estimated to be 106,760 [5]. Accordingly, 13,626 patients registered by this program corresponded to approximately 13 % of the whole population affected by gastric cancer in Japan. Even though these patients may not represent the average features of gastric cancer found in this country, this report is considered to have analyzed the largest number of patients for the past 10 years, clarifying the trends of gastric cancer in Japan. Just for reference, the proportion of patients registered in the nationwide registry of other organs of all patients diagnosed were 6 % in colon cancer, 24 % in esophageal cancer, 25 % in liver cancer, and 26 % in lung cancer, respectively [6].

The reliability of the results in this report depends on the quality of data accumulated in the JGCA database. Because of the complexity of the JGCA staging system, the error checking system on the data entry screen did not function completely. In several categories such as lymph node metastasis (N), the JGCA system could not be converted to the TNM system automatically. Therefore, the registration committee had to make great efforts to confirm raw data sent to the data center from the participating hospitals.

As compared with our archived data of 12,004 patients treated in 2001 [1], the proportion of early cancer declined from 51.2 % to 49.7 % [pT1 (M) cancer, 27.4 % to 25.6 %, and pT1 (SM) cancer, 23.8 % to 24.1 %], suggesting that an increasing number of patients with mucosal cancer were sent for endoscopic treatment. These data suggest that we should start to register gastric patients treated with endoscopic mucosal resection (EMR) and/or endoscopic submucosal dissection (ESD) as soon as possible. The surgical mortality within 30 days significantly improved, from 0.6 % to 0.48 % (*P* < 0.001). Just for reference, it was 4.0 % in 1963 and 1.0 % in 1991 [7], Moreover, the nationwide database of gastrointestinal surgery in 2008 showed that was 0.2 % in gastrectomy and 0.4 % in total gastrectomy [8].

**Fig. 18:**
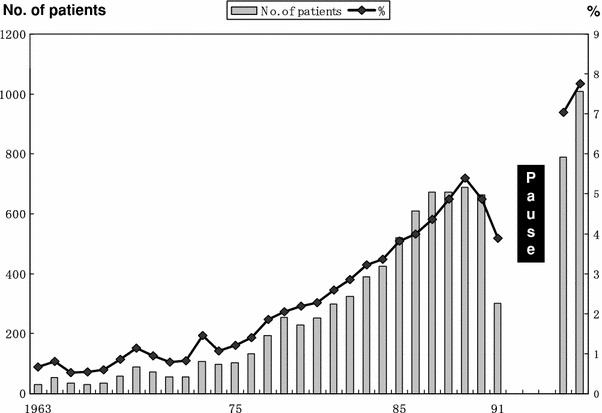
Chronological change of gastric cancer patients older than 80 years. The nationwide registry was suspended for a decade from 1992

Accordingly with the rapidly aging society in Japan, the proportion of patients more than 80 years old continued increasing (Fig. 18): it was 0.7 % in 1963, 4.9 % in 1990, 7.0 % in 2001, and 7.8 % in 2002, respectively. Although the risk for surgery increases in elderly patients who have comorbidities, evaluations of risk can allow interventions that may decrease morbidity and mortality. Appropriate treatments should be offered to the elderly. However, these data have the intrinsic weakness of being retrospectively collected 7 years after surgery. Unfortunately, we in Japan continue to have a legal difficulty in registering personal information, which is essential for long-term and prospective follow-up. The overall follow-up rate in our program was 83.5 %. In other words, the outcome of 17.5 % of the patients is unknown. The proportion of patients who were lost to follow-up in the Japanese nationwide registry of colon cancer, liver cancer, and thyroid cancer was 19.6 %, 25.8 %, and 20.6 %, respectively [6]. Rules and regulations regarding handling of these data will have to change radically to overcome the issue of accuracy and reliability of the nationwide registry in Japan, and this could be out of the hands of the surgeons who have contributed to the best of their abilities to gather these data. On the other hand, the Japanese Association of Clinical Cancer Centers, consisting of 25 cancer center hospitals, reported that their follow-up rate was 98.5 %, and 5YEARS of 9,980 patients who underwent surgery from 1997 to 2000 were 90.4 % for TNM stage I, 67.8 % for stage II, 43.3 % for stage III, and 9.3 % for stage IV, respectively [9]. When the patients with gastric cancer had a medical examination in clinical cancer centers, they registered the place where their family records were registered, and office workers of the clinical cancer centers confirmed regularly their safety from the family registration; this was the reason for the extremely high follow-up rate. In the current analyses, 5YEARS in stage IV patients was 15.2 %. We might have overestimated our 5YEARS in stage IV patients, but we found that our follow-up rate increased as the stage advanced; the follow-up rate of stage IV patients was 90.4 %. These data suggest that the lower follow-up rate may not have had serious effects on 5YEARSs in our program. Although, the correlation between follow-up rate and survival rate is complicated, our follow-up system needs to be improved if we are to evaluate the survival rates more accurately.

Cytological examination was conducted in 3,481 (59.4 %) of 5,857 patients with T2, T3, or T4 cancer. The 5YEARS of CY1 patients was 12.3 % and their 5YEARS was as poor as that of patients with peritoneal metastasis. Although this examination was not carried out commonly in the days of 2002, it could still be regarded as a significant and independent prognostic factor from the data that were available. These findings further support the need for staging laparoscopy for accurate preoperative staging in patients with advanced gastric cancer.

JGCA restarted a nationwide registration from 2008. The object of the new nationwide registry was primarily to calculate the stage-specific 5YEARSs among patients who underwent gastrectomy. Therefore, the structure of the database was required to be simple, and the number of registration items was kept to a minimum. Undoubtedly, the next objective would be to collect and analyze data of patients with inoperable disease, remnant gastric cancer, gastrointestinal stromal tumor, malignant lymphoma of the stomach, and other entities that were excluded in the current project. We also began to register patients who were treated by EMR/ESD by adding additional items and updating data entry software from 2011.

We hope that this report will be useful when surveying trends and changes in the clinical practice and treatment results of gastric cancer in Japan. Details of the individual data presented in this report will soon become available for scientific and clinical research with the permission of the registration committee. In addition, most of the surgical and pathological data could easily be transferred to the international database in the near future for various analyses. The registration committee will continue the efforts to improve the registration system, ultimately to collect meaningful annual data.

## Appendix: Participating hospitals

Data of gastric cancer patients in this report were collected from the surgical or gastrointestinal departments of the following 208 hospitals (in alphabetical order): Akashi Municipal Hospital, Aomori City Hospital, Asahikawa Medical University Hospital, Cancer Institute Hospital, Chiba Cancer Center, Chiba University Hospital, Dokkyo Medical University Hospital, Ebina General Hospital, Fuchu Hospital, Fujita Health University (Banbuntane Houtokukai Hospital), Fukaya Red Cross Hospital, Fukui Red Cross Hospital, Fukuoka University Chikushi Hospital, Fukuoka University Hospital, Fukushima Medical University Hospital, Gifu Prefectural General Medical Center, Gifu University Hospital, Gunma Prefectural Cancer Center, Gunma University Hospital, Hakodate Goryoukaku Hospital, Hakodate Municipal Hospital, Hamamatsu University School of Medicine, University Hospital, Handa City Hospital, Health Insurance Hitoyoshi General Hospital, Higashiosaka City General Hospital, Himeji Central Hospital, Hiroshima City Asa Hospital, Hiroshima City Hospital, Hiroshima Prefectural Hospital, Hiroshima Red Cross Hospital and Atomic-bomb Survivors Hospital, Hiroshima University Hospital, Hitachi General Hospital, Hokkaido University Hospital, Hoshigaoka Koseinenkin Hospital, Hospital, University of the Ryukyus, Hyogo Cancer Center, Hyogo Prefectural Nishinomiya Hospital, Ibaraki Prefectural Central Hospital, Ibaraki Seinan Medical Center Hospital, Ishikawa Prefectural Central Hospital, Iwate Medical University Hospital, Iwate Prefectural Central Hospital, Iwate Prefectural Kamaishi Hospital, Izumi Municipal Hospital, JA Hiroshima Kouseiren Hiroshima General Hospital, Japanese Red Cross Medical Center, Jikei University School of Medicine, Jikei University Aoto Hospital, Juntendo University Juntendo Hospital, Jusendo Medical Hospital, Kagawa Prefectural Central Hospital, Kagawa Medical University Hospital, Kakogawa Municipal Hospital, Kanagawa Cancer Center, Kanazawa University Hospital, Kansai Electric Power Hospital, Kansai Rousai Hospital, Kawasaki Medical School Hospital, Keio University Hospital, Keiyukai Sapporo Hospital, Kimitsu Chuo Hospital, Kinki Central Hospital, Kinki University Hospital, Kiryu Kosei General Hospital, Kitakyushu Municipal Medical Center, Kobe Century Memorial Hospital, Kobe City Medical Center General Hospital, Kouchi Medical School Hospital, Kumamoto Medical Center, Kumamoto Regional Medical Center, Kumamoto University Hospital, Kurashiki Central Hospital, Kurobe Kyosai Hospital, Kuroishi General Hospital, Kurume University Hospital, Kushiro Rosai Hospital, Kyorin University Hospital, Kyoto Second Red Cross Hospital, Kyoto University Hospital, Kyushu University Hospital, Matsushita Memorial Hospital, Matsuyama Red Cross Hospital, Misawa City Hospital, Mitoyo General Hospital, Miyagi Cancer Center, Mizushima Kyodo Hospital, Muroran City General Hospital, Musashino Red Cross Hospital, Nagahama City Hospital, Nagano Municipal Hospital, Nagano Red Cross Hospital, Nagaoka Chuo General Hospital, Nagasaki Municipal Hospital, Nagoya University Hospital, Nakagami Hospital, Nanpuh Hospital, Nara Medical University Hospital, Nara Hospital, Kinki University Faculty of Medicine, National Cancer Center Hospital, National Defense Medical College Hospital, NHO Ciba Medical Center, NHO Kasumigaura Medical Center, NHO Kyushu Cancer Center, NHO Osaka Medical Center, NHO Sendai Medical Center, NHO Shikoku Cancer Center, NHO Tokyo Medical Center, NHO Yokohama Medical Center, Nihon University Itabashi Hospital, Nihon University Surugadai Hospital, Niigata Cancer Center Hospital, Niigata City General Hospital, Niigata Prefectural Shibata Hospital, Niigata Prefectural Yoshida Hospital, Niigata University Medical and Dental Hospital, Nippon Koukan Hospital, Nippon Medical School Chiba Hokusoh Hospital, Nippon Medical School Hospital, Nishi-kobe Medical Center, NTT West Osaka Hospital, Obihiro Tokushukai Hospital, Oita Red Cross Hospital, Oita University Hospital, Okayama University Hospital, Okitama Public General Hospital, Onomichi Municipal Hospital, Osaka City University Hospital, Osaka General Medical Center, Osaka Kouseinenkin Hospital, Osaka Medical Center for Cancer and Cardiovascular Diseases, Osaka Medical College Hospital, Osaka Police Hospital, Osaka Red Cross Hospital, Osaka Seamen’s Insurance Hospital, Osaka University Hospital, Otsu Municipal Hospital, Otsu Red Cross Hospital, Rinku General Medical Hospital, Sado General Hospital, Saga University Hospital, Saiseikai Chuwa Hospital, Saiseikai Fukuoka General Hospital, Saiseikai Kumamoto Hospital, Saiseikai Niigata Daini Hospital, Saiseikai Noe Hospital, Saiseikai Utsunomiya Hospital, Saitama Medical Center, Saitama Medical Center Jichi Medical University, Saitama Red Cross Hospital, Saitama Social Insurance Hospital, Saku Central Hospital, Sapporo City General Hospital, Sapporo Medical Center, Sapporo Medical University Hospital, Sapporo Social Insurance General Hospital, Sayama Hospital, Seirei Hamamatsu General Hospital, Shakaihoken Kobe Central Hospital, Shiga University of Medical Science Hospital, Shimonoseki City Central Hospital, Shinnittetsu Yahata Memorial Hospital, Shinshu University Hospital, Shizuoka Cancer Center, Showa Inan Hospital, Showa University Northern Yokohama Hospital, Showa University Toyosu Hospital, Social Insurance Central General Hospital, Social Insurance Kinan Hospital, Southern Tohoku General Hospital, St. Luke’s International Hospital, St. Marianna University School of Medicine Yokohama City West Hospital, Suita Municipal Hospital, Sumitomo Hospital, Suwa Red Cross Hospital, Takeda General Hospital, Tochigi Cancer Center, Toho University Ohashi Medical Center, Tohoku University Hospital, Tokushima Municipal Hospital, Tokushima Prefectural Central Hospital, Tokushima University Hospital, Tokyo Medical University Ibaraki Medical Center, Tokyo Metropolitan Bokutoh Hospital, Tokyo Metropolitan Cancer and Infectious Disease Center Komagome Hospital, Tokyo Women’s Medical University (Institute of Gastroenterology), Tokyo Women’s Medical University Hospital, Tokyo Women’s Medical University Medical Center East, Tonami General Hospital, Toranomon Hospital, Tottori Municipal Hospital, Toyama Prefectural Central Hospital, Toyama University Hospital, Toyohashi Municipal Hospital, Tsuchiura Kyodo General Hospital, Tsukuba University Hospital, University Hospital Kyoto Prefectural University of Medicine, University of Fukui Hospital, University of Miyazaki Hospital, University of Yamanashi Hospital, Wakayama Medical University Hospital, Yamachika Memorial General Hospital, Yamagata Prefectural Central Hospital, Yamagata University Hospital, Yamaguchi Rousai Hospital, Yamanashi Prefectural Central Hospital, Yao Municipal Hospital, Yodogawa Christian Hospital, Yokohama City University Hospital, Yokohama City University Medical Center, and Yuri Kumiai General Hospital.
